# Expanding the Concept of Precision Nursing from a Caring Science Perspective, with Clinical Examples from a Swedish Emergency Care Context

**DOI:** 10.3390/healthcare14060789

**Published:** 2026-03-20

**Authors:** Sofia Almerud, Lise-Lotte Augustine, Anna Bennesved, Ingrid L. Gustafsson, Ann-Therese Hedqvist, Jeanette Lindahl, Tünde Mako, Johanna Rosenqvist, Anders Svensson, Emma Westin, Sara C. Wireklint, Carina Elmqvist

**Affiliations:** Centre of Interprofessional Collaboration within Emergency Care (CICE), Region Kronoberg and Linnaeus University, 351 95 Växjö, Sweden; lise-lotte.augustine@kronoberg.se (L.-L.A.); anders.svensson@lnu.se (A.S.);

**Keywords:** emergency care, caring, patient centered care, precision medicine, precision nursing

## Abstract

The purpose of both precision medicine and precision nursing is to improve patient outcomes. This theoretical article is designed as a conceptual and position paper situated within caring science, with a specific focus on understanding precision nursing across diverse clinical contexts. Rather than presenting empirical findings, the paper synthesizes theoretical perspectives, caring science foundations, and selected scholarly literature. In this position paper, we seek to expand the concept of precision nursing from a caring science perspective with clinical examples, vignettes, from an emergency care context. Precision medicine can be viewed as an effort to truly individualize a treatment and make it as accurate and effective as possible. While the focus on measurable outcomes saves lives, it also carries the risk of narrowing attention to what can be observed and quantified. These visible clinical markers represent only part of what matters in care. To get the full picture of a patient and their treatment, caring must serve as the foundation for precision nursing, as it is caring that ensures that technological advancements remain aligned with individual patient needs. Precision medicine and precision nursing may offer direction, but to provide meaning to the concepts, a grounding in caring science is provided in this study.

## 1. Introduction

The purpose of both precision medicine and precision nursing is to improve patient outcomes. This theoretical article is designed as a conceptual and position paper situated within caring science, with a specific focus on understanding precision nursing across diverse clinical contexts. Rather than presenting empirical findings, the paper synthesizes theoretical perspectives, caring science foundations, and selected scholarly literature.

The clinical cases used throughout the paper are presented as illustrative vignettes, highlighting challenges such as autonomy, urgency, communication, emotional needs, and ethical complexity. They do not constitute empirical data, nor were they collected through a systematic research process. Instead, they are grounded in the authors’ clinical experience and supported by established descriptions in the literature. Their purpose is to clarify theoretical concepts and demonstrate how principles of precision nursing and caring science may unfold in real clinical situations. These vignettes serve as a starting point for our discussion of precision nursing from a caring science perspective.

To this end, precision medicine focuses on biologically optimized treatments combined with precision nursing, which strives to ensure that the patient receives treatments that they can handle. For example, a nurse can use genetic data to inform treatment options (precision medicine) while also considering the patient’s preferences and values (nursing), in order to ensure the chosen treatment is acceptable and feasible for the patient [1]. Caring refers to the compassionate, relational, and humanistic aspect of that interaction. It encompasses empathy, attentiveness, and a focus on the patient as an individual. In practice, the assessment of a patient’s condition involves both physiological evaluation and an understanding of the person’s lived experience and subjective care needs. These aspects are inseparable. Nursing encompasses both the science of medical care and the art of human caring. The aim of this paper is to expand the concept of precision nursing from a caring science perspective, with clinical examples from a Swedish emergency care context.

## 2. Theoretical Framework

Precision medicine is defined as an approach that uses various information about a person’s individual differences in terms, e.g., of their genetics, environment, and lifestyle, in order to customize treatment and prevention strategies to provide the right care for the right person at the right time [2]. Precision medicine tailors prevention and treatment strategies to individual variability in treatment response. It leverages large-scale biological data (e.g., genomics, proteomics, and metabolomics), advanced data categorization, mobile health technologies, and computational tools for analysis [1]. In the literature, precision nursing is often equated with precision medicine. Both are rooted in biomedical science and technology and aim to improve patient outcomes [1]. However, precision nursing can also be grounded in caring science, a holistic approach that values the patient’s personal experience. Precision medicine requires advanced tools and technologies for data analysis and interpretation, whilst caring science relies more on communication skills, empathy, and understanding the patient’s perspective. The goal of caring science is to alleviate suffering and create conditions for well-being [3,4]. It constitutes an important component and resource in delivering high-quality nursing in accordance with the principles of precision nursing. Nurses are professionally obligated to promote a supportive care environment, as outlined in the ethical code of the International Council of Nurses [5].

## 3. Vignettes from Various Emergency Care Contexts

### 3.1. An Encounter in the Ambulance Service

In Sweden, ambulances are staffed by at least one nurse, most of whom have specialized training in ambulance care. Throughout this text, the term ‘nurse’ will be used to refer to this role.

Carl, 78, has a medical history of heart failure, chronic obstructive pulmonary disease (COPD) and mild cognitive impairment. He lives in a rural area, 14 min from the nearest hospital. When Carl appears to be experiencing acute respiratory distress, a neighbor calls for an ambulance. Upon arrival, the nurse finds Carl anxious and hesitant to go to the hospital. The initial clinical assessments are as follows—SpO_2_: 78% on 1 L home oxygen; respiratory rate: 38/min; pulse: 138 bpm; BP: 105/70 mmHg.

Rather than immediately enforcing a treatment plan, the nurse initiates a calm, face-to-face dialog, exploring not only Carl’s clinical symptoms but also his emotional and existential concerns. Carl expresses a fear of hospitalization and worries about burdening his family, especially following the recent death of his wife. By first acknowledging his emotional and existential distress, the nurse helps reduce anxiety-driven dyspnea and thereby creates conditions for a more accurate assessment and care.

Using the ambulance’s digital systems, the nurse accesses Carl’s electronic health record (EHR), enabling them to make precision-informed care decisions: Oxygen therapy is cautiously titrated, avoiding high-flow delivery due to COPD-related CO_2_ retention risk. Medication management involves low-dose sublingual nitroglycerine, adjusted for Carl’s heart failure and blood pressure. Avoidance of sedatives based on previous EHR-documented episodes of delirium.

In this vignette the caring science perspective is highlighted by simultaneously acknowledging Carl’s existential concerns and individual needs to meet him as a whole person and not merely a set of clinical symptoms. The caring science perspective fosters trust and supports shared understanding in a moment when the patient might otherwise feel powerless. This interaction reflects core principles: the nurse listens attentively and acknowledges Carl’s full narrative and individual needs.

### 3.2. Encounters at the Emergency Department

In Sweden, patients can self-refer to the emergency department (ED). Upon arrival they are assessed by a nurse and subsequently triaged.

Wendy, 86, has COPD. She arrives at ED on a Sunday, accompanied by her concerned son. He has noticed that she is unusually tired and believes hospitalization is necessary. When the nurse asks how she feels, Wendy acknowledges that she may have missed a dose or two of her daily maintenance inhaler. While speaking with her, the nurse assesses her resting respiratory rate, while also noting her posture, lip color, skin, voice, and vital signs. The nurse’s findings include fatigue, forward-leaning posture, pale lips, dry skin, a weak voice, and low oxygen saturation, all of which suggest a COPD exacerbation. Without further delay, regardless of the exact cause, the nurse rapidly administers inhalation and oxygen therapy to ease Wendy’s breathing, followed by routine tests and documentation.

Nouriah, a mother with limited proficiency in Swedish, calls the healthcare advisory line on behalf of her 12-year-old daughter, Maryam, who has a sore throat and mild fever. Due to communication difficulties, the healthcare advisory line nurse advises a visit to the ED as a precaution. They arrive just before 3 p.m. and wait nearly an hour before triage. The nurse interviews them, with Maryam helping to translate when questions are aimed at her mother. After the interview and physical examination, the nurse finds no signs of a life threatening or severe condition. Maryam does however have a right-sided soreness and a slight swelling in her throat and a mild fever. The triage scale Rapid Emergency Triage and Treatment System (RETTS) places her between the two lowest priority levels, suggesting her treatment can be managed at home. The local decision-making instrument for levels of care also recommends either self-care or care within 24 h. According to local guidelines, primary care (PC) is responsible for ear–nose–throat (ENT) related cases. Despite this, the nurse feels an ENT evaluation is appropriate, due to the language barrier and the family’s proper use of the health care system. The ENT physician declines to see the patient, citing PC responsibility. The nurse contacts the family’s PC clinic, but they are closing for the day and cannot offer Maryam an appointment. The out-of-hours PC clinic requires patients to book appointments themselves, so the nurse’s request for an appointment is denied. The nurse provides oral and written self-care instructions and contact details for the out-of-hours PC clinic. The family heads home, leaving the nurse to reflect on how the system often overlooks non-medical factors in care decisions.

In this vignette, the caring science perspective is highlighted by the nurses’ compliance to the needs demonstrated by the patients. By doing so, the nurse provides a sense of safety for both patients and confidence for further treatment. These patients arrive at the ED as so-called walk-ins and are assessed in the triage, which is a highly intense and stressful environment. The triage is to be considered the intersection between medical and nursing care, a place where medical knowledge is enhanced by the caring science perspective while simultaneously regulated by organizational care agreements regarding the triage patient’s health, on display with all its vulnerability, anxiety, and suffering, which the nurse needs to address with sincerity and humanity.

### 3.3. Encounters in Hospital Wards

“She doesn’t want anything, it’s impossible to mobilize her, she’s just angry and grumpy all the time. It’s just difficult to get through to her,” a younger colleague reported to the senior evening nurse. Eliza, 59, had suddenly fallen ill 45 days earlier. Once a healthy, working professional, she now lay powerless behind a closed door, staring at the ceiling. It was midsummer, and she had not showered or felt the sun on her face or wind in her hair for 46 days. Eliza’s husband was only present during the initial critical phase of her illness; since then, COVID-related visitation restrictions limited her contact with him and their children. For nearly 30 ward rounds, Eliza’s extreme situation went unacknowledged. The focus remained on clinical values, diagnostics, and treatment. Though her parameters normalized, she remained immobile and disengaged. Plans for rehabilitation seemed unrealistic. The person behind the test results was overlooked, and Eliza lacked the strength to express her own needs. When the senior nurse zeroed in on her care, they, too, were met with disinterested replies from the patient (“I don’t want to”, etc.). Yet this nurse remained present and kept listening to Eliza’s complaints and questions: “I can’t do it myself.” “Can I really shower with these tubes?” The nurse addressed her concerns with answers, and the very next day, Eliza’s hair was washed. Her central line was removed after she questioned during rounds why it had yet to be removed. On the third day, she said, “I’d like to go home to my garden—but I don’t have the strength to sit in the car.” Arrangements were made to transport her in a supine position, and within a week, she was transferred to rehab. With the support of an experienced nurse, Eliza’s voice and the nursing perspective were brought into the clinical discussion, helping the team better meet her needs. In daily rounds, the composition of the participating healthcare professionals and their experiences vary, and this often affects the quality of care. Could rounds be designed to consistently address patient needs, regardless of team makeup, so that stories like Eliza’s aren’t repeated?

A young man, Brian, 31 is admitted to the intensive care unit (ICU) with ketoacidosis in a critically unstable condition that requires immediate intervention. Though he has a history of self-harm and multiple hospitalizations, this is his first ICU admission. After 12 h of treatment, his condition improves, but he remains hypertensive and tachycardic and grows increasingly anxious, expressing a desire to leave the ICU. Despite sedatives and sleep aids, he remains restless and eventually requires IV sedation to rest, resulting in fatigue and the delay of his transfer to a general ward. The following night, a nurse engages Brian in conversation. He opens up about his ongoing anxiety and reaches out physically, which the nurse interprets as a need for contact. The nurse gently holds his hand and offers to massage his hands, feet, and lower legs, which the patient welcomes. During the massage, his vital signs stabilize, and he falls asleep. The following day, Brian was discharged from the ICU.

In this vignette, the caring science perspective is highlighted by viewing the caring encounter as essential, making it possible to see the whole person, and thereby provide care that not only treats the diagnoses and physical symptoms but also strengthens the patients’ humanity, dignity and participation.

### 3.4. An Encounter in the Pediatric Emergency Department

The pediatric ED treats children and adolescents of all ages. Some have had multiple encounters with the healthcare system, while for others it is their first time. One such patient is Tara, 6, who has come to the pediatric ED due to a fever and breathing difficulties. There is a suspicion of pneumonia, and in order to make a diagnosis and begin appropriate treatment, blood samples and an X-ray examination are required. The nurse in the ED has spoken with Tara and her father and learned that Tara has previously had blood samples taken at her PC clinic. However, it was an unpleasant experience, and Tara is feeling anxious about today’s procedure. She tries to hide her arms under her shirt and keeps repeating, “No, I don’t want to,” over and over again. Using a stuffed animal, Tara is shown how the blood sample will be taken. She agrees to the application of numbing cream and is told she can choose whether to watch the procedure or focus on something else. The nurse reassures her that all emotions are okay—even crying or screaming, if it helps. Tara opts for distraction and sits on her father’s lap for comfort. While they blow soap bubbles, the blood sample is taken successfully. Afterward, she proudly shows off her emoji-covered plaster. She then watches a video about chest X-rays, which she does not find to be scary, and calmly follows staff into the imaging room. The nurse documents the experience in Tara’s medical record to guide her future care.

In this vignette, the caring science perspective is highlighted by the nurse’s ability to meet Tara, involve her, and then base the care on what Tara herself believes is best for her. This increases her sense of control and reduces fear. This can only be achieved by listening to Tara, her previous experiences, and her current wishes.

## 4. Reflection and Discussion

Emergency care must adapt to an aging population and increasingly diverse care needs, and precision nursing from a caring science perspective offers a model that integrates, e.g., technology, ethics, and relational care. This approach does not only improve outcomes, but also honors the patient as a person. In dynamic emergency care, this balance is both an art and a necessity.

The first case illustrates how patient-specific data can guide safe, effective, and individualized care decisions in the prehospital setting. Carl’s case raises important ethical considerations: Can his preference to stay home be honored without compromising safety? Does his cognitive impairment affect his decision-making capacity? How should the nurse balance benefit and respect for Carl’s autonomy? These questions are addressed through a transparent and inclusive dialog involving Carl, his relatives and other healthcare professionals. This collaborative decision-making process aligns with ethical nursing practice, ensuring decisions are both clinically and ethically defensible. A key aspect of this case is the nurse’s ability to prioritize values in a complex and time-sensitive situation. By allowing Carl a few moments before initiating medical treatment, the nurse created space for calm and presence. This pause enabled a more nuanced assessment of which symptoms could be addressed through relational and emotional support and which required pharmacological intervention. The ability to distinguish between what must be treated medically and what can be addressed through caring presence is a core competence. In the ambulance service, precision nursing from a caring science perspective may involve integrating clinical guidelines with real-time data, electronic health records, and rapid yet meaningful communication focused on the patient’s preferences and lived experience. Unlike traditional guidelines that revolve around standardized responses, precision nursing from a caring science perspective calls for adaptability and responsiveness tailored to individual needs. Ambulance services often act as the first point of contact for patients with acute or complex health issues. Traditionally, the focus has been on rapid assessment, stabilization, and transport. However, rising patient complexity—particularly among elderly patients with multimorbidity—demands more nuanced approaches [6]. Through its emphasis on individualized assessments, continuous quality improvement, and ethical responsiveness, caring science offers a framework for meeting these emerging demands [7]. As an example, Holmberg’s [8] EXPAND model illustrates how prehospital emergency nursing integrates biomedical and existential dimensions through lifeworld-led interpretation. In this view, caring science is not separate from clinical judgment, but embedded in how signs are interpreted and actions are taken. The act of caring is thus not something that occurs after a diagnosis; it is interwoven with how knowledge is produced, how risks are assessed, and how treatment is initiated.

Precision nursing from a caring science perspective has not traditionally been a focus in EDs, where attention has often centered on the lack of nursing care [9,10]. However, triage (sorting patients based on urgency) can be seen as a form of precision nursing. It addresses the mismatch between patient volume and available resources, aiming to deliver the right care at the right time [11]. Triage, from the French trier (‘to sort’), is the process of prioritizing ED patients based on their main complaint and symptoms, in order to identify critical cases and determine wait times [12]. In Sweden, most EDs use the Swedish triage system RETTS, typically managed by RNs, in addition to the vital signs required by the system. According to this model, triage often includes early interventions like blood tests, ECGs, administration of analgesics and X-rays to streamline patient flow [13]. Triage also offers RNs a chance to practice precision nursing from a caring science perspective, allowing them to draft reports quickly and provide customized care, even in brief encounters [14]. This was highlighted by RNs when they described how they allocated time they did not actually have in the triage, in favor of the patient. In doing so, they got the time they needed to establish a caring relationship in which the RN and the patient could reach a mutual understanding about the triage decision, even if it ended in the RN advising self-care to the patient [13].

In the hospital ward, medical technology need not strip patients of their individuality, subjectivity, or dignity. The issue lies not in the technology itself, but in how it is used—whether care remains humane and holistic, or becomes purely technical and impersonal [15]. Precision nursing from a caring science perspective requires genuine curiosity, professional humility, and a willingness to share power with the patient and their close network. Our most immediate tools are ourselves: our voices, our hands, our presence. Caring touch is a natural tool for healthcare professionals, and touch can provide a better assessment of a patient’s condition. However, it is important that healthcare professionals reflect on how such touch is perceived by the patient, because caring touch can be both positive and negative [16].

Reconnecting the ‘art’ and the ‘act’ of nursing is key to ensuring treatment based on patients’ needs and diffusing the tension between technical care and human connection. This integration is the core of precision nursing from a caring science perspective—navigating the ambiguity between technology and humanity to deliver genuine care; c.f., [15]. Intertwining ‘the doing’ and ‘the being’ alleviates the sense among healthcare professionals that they are split between providing care and responding to patients’ care needs [17].

In the pediatric clinical setting, precision nursing from a caring science perspective is required in order to ensure that medical procedures are carried out on the child’s own terms. Children often experience anxiety in healthcare settings, which can hinder cooperation during procedures. Maintaining calm and reducing fear is essential for building trust [18]. During needle-related procedures, children may fear not only the pain, but also the unfamiliar environment, unknown staff, and uncertainty [19]. In Tara’s case, while the procedure was medically necessary, her emotional needs also needed to be addressed. If she had felt powerless or ignored, her resistance could have escalated, potentially leading to the use of physical restraint, which can leave children feeling ashamed and powerless [20]. Such experiences may cause a child to develop a long-term fear, such as a phobia of needles, and deter them from seeking care in the future [21]. In all procedures, the best interests of the child must be prioritized over the interest of their parents or the organization. Hence, care must be tailored according to each child’s unique experiences, abilities and wishes. Healthcare professionals must reduce the power imbalance between themselves and their young patients by fostering collaboration and listening to children. A child-centered approach that considers both the child’s own views and the perspectives of adults [21] is essential. These perspectives should be applied in tandem and guide decisions about what is best for the child in both the short and long term.

Ethical challenges are inherent in, e.g., emergency care, particularly when balancing clinical urgency with individual needs. Vulnerable patients—such as those with language barriers, cognitive decline, or mental illness—may struggle to express their needs [22]. Unfortunately, many patients and their family members find EDs to be stressful, overcrowded, and anxiety-provoking. They often entail long wait times and a sense of vulnerability, fear, dependence, and lack of control. The care environment plays a crucial role in shaping these emotional responses and directly influences the patient’s experience—which can range from calm and secure to chaotic and distressing. A supportive care environment is one that conveys a welcoming atmosphere characterized by safety and security, ease of orientation, opportunities for choice and control, and respect for privacy and confidentiality [23].

Inter-organizational collaboration in the treatment of patients with complex care needs highlights the importance of caring in practice [24]. Research illustrates how seamless care cannot be achieved through clinical routines and precision medicine alone. Instead, adaptability, communication, and collaborative practices grounded in the patient’s lived experience are essential. Patients with multiple chronic conditions and intersecting social needs often navigate fragmented systems in which standardized interventions may fail to capture what truly matters to them. Peters et al. [25] underscore that patients with complex healthcare needs highly value continuity, relational trust, and shared decision-making—elements often overlooked when organizational systems prioritize efficiency over individualization. Similarly, Louw et al. [26] point to the need for context-sensitive approaches that move beyond checklists and indicators.

A systematic review by Ebrahimi et al. [22] highlights difficulties related to interprofessional coordination, unclear roles, and limited engagement with the patient perspective. These findings align with Hedqvist et al. [24], who found that while digital solutions like video-conferencing in care planning can improve access and coordination, the risk remains that technology may replace genuine human interaction, rather than supporting it. In these contexts, healthcare professionals must remain vigilant to ensure that the individual patient’s story remains central to the process of caring for that unique person.

We understand precision medicine as well as precision nursing as a commitment to truly individualize a treatment and make it as accurate and effective as possible. The focus on effect and measurable outcome saves lives, but with a risk of losing sight of the person as a whole.

Complaints about being torn between care and technology miss the point entirely; both are equally vital. Advanced tools must be balanced with compassionate, comprehensive care, harmonizing objective data with subjective experience c.f., [15].

In emergency care, there is a risk that urgency may eclipse the patient’s involvement in decision-making, despite the fact that patients with urgent needs highly value continuity, trust and shared decision-making [18]. For instance, an elderly patient with heart failure may refuse hospitalization in favor of staying at home, thus requiring the nurse to weigh their clinical judgment against their respect for the person’s autonomy. This tension is echoed in a study by Bennesved et al. [27] on ambulance clinicians’ perceptions of older patients’ self-determination. While ambulance clinicians acknowledge the principle of autonomy, decisions are often shaped by workload, perceived risk, and expectations from other healthcare professionals. These findings highlight the importance of ethical reflections and structural support for the provision of precision caring in emergency care real-time settings [27]. Using the ‘Art of Caring’ model as an approach to ethical reflections is one way to meet the challenges various healthcare professionals encounter within emergency care, in order to find a balance between giving and receiving [17].

## 5. Conclusions and Implications

If precision medicine as well as precision nursing is the visible tip of the iceberg, an enormous amount of important information about the sick person—all their experiences, possibilities, obstacles, expectations, and hopes, remains invisible below the surface. In clinical practice a caring science perspective combines both what is visible and not visible. As an example, not all patients with a disease feel ill or experience suffering. Some patients may experience greater illness and suffering as the result of a specific treatment or lack of existential support. There is a wealth of possible data-informed, person-specific strategies that combine clinical insights with compassionate, context-aware engagement.Precision nursing cannot be reduced to technical accuracy alone; it requires a holistic and ethically grounded approach that honors the uniqueness of each patient in complex care environments. An expanded view of precision nursing from a caring science perspective is based on the whole picture, not only what meets the eye. Thus, caring for each individual person on their own terms ought to be the essence of precision nursing. Precision may guide clinical decisions, but caring imbues them with meaning.

This paper may serve as a foundational resource for future research seeking to broaden the perspectives of the nursing profession regarding the concept of precision nursing and its clinical significance. The motivation for expanding the conceptualization of precision nursing from a caring science perspective is to reinforce the professional role of nurses and to advance the field of clinical nursing research. Furthermore, this work may provide a conceptual basis for the development of a pilot program aimed at strengthening academic competence more broadly.

## Data Availability

No new data were created or analyzed in this study. Data sharing is not applicable to this article.
